# Staged Management of Vascular Occlusion With Ellansé (Polycaprolactone): A Modified Delphi Expert Consensus

**DOI:** 10.1093/asjof/ojag167

**Published:** 2026-09-30

**Authors:** Francisco de Melo, Shang-Li Lin, Franco Vercesi, Gabriel Aribi, Erick Santaella-Sosa, Filis Demirgean, Mohamed Abdelrahman, Ryaan Sultan, Mariyah Yazdani

## Abstract

**Background:**

Vascular occlusion (VO) is a rare but serious complication of dermal filler injection. Current management protocols are derived primarily from hyaluronic acid fillers and rely on enzymatic degradation with hyaluronidase. Ellansé (PCL-CMC) is a poly-ε-caprolactone (PCL)-based filler composed of PCL microspheres suspended in a carboxymethyl cellulose (CMC) gel carrier, neither of which is a substrate for hyaluronidase; a product-specific management approach is therefore required.

**Objectives:**

To develop expert consensus recommendations for the recognition and management of VO associated with PCL-CMC.

**Methods:**

A targeted literature review (PubMed, January 2009–August 2025), in vitro rheometry and flow testing, and a three-round modified Delphi consensus process involving seven international experts were used to develop a staged management algorithm.

**Results:**

At 1:5 dilution, PCL-CMC viscosity decreased approximately 80-fold to near-aqueous levels; hyaluronidase solution conferred no additional benefit over saline alone. A full consensus or a consensus based on majority of the experts consensus (≥80%) was achieved on the majority of statements, including CMC-mediated obstruction as the primary mechanism, high-volume saline flushing with mechanical dispersion as first-line therapy, and retention of hyaluronidase in a supportive role.

**Conclusions:**

This consensus-derived algorithm prioritizes reperfusion through dilution and mechanical dispersion rather than enzymatic degradation. Prospective clinical validation is required.

**Level of Evidence: 5 (Therapeutic):**

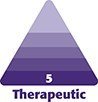

The injection of dermal fillers is one of the most commonly performed aesthetic procedures globally, with more than 6.3 million procedures performed in 2024.^1^ Although generally safe, vascular occlusion (VO) remains a rare but serious complication that can lead to tissue ischemia, skin and other soft tissue necrosis, permanent scarring, and irreversible visual loss when retinal arteries are involved.^2-5^ The increasing global adoption of non-HA biostimulatory fillers has heightened the need for product-specific safety protocols that account for differing material properties and degradation pathways.

Ellansé (AQTIS Medical BV, Utrecht, The Netherlands) is a poly-ε-caprolactone (PCL)-based dermal filler composed of smooth PCL microspheres (25-50 µm; 30% by volume) suspended in a carboxymethyl cellulose (CMC) gel carrier (70% by volume).^6^ The product provides immediate volumization with longer-term connective tissue stimulation.^7^ Global pharmacovigilance data suggest a very low reported incidence of VO with PCL-CMC (0.0005%);^8^ however, its distinct physicochemical properties—a nonhyaluronidase-degradable carrier gel and slowly resorbing polymeric microspheres—necessitate a product-specific management approach.^6^

Current VO guidance is largely derived from hyaluronic acid (HA) fillers, where high-dose pulsed hyaluronidase is central to management.^2,9^ As neither PCL nor CMC is a substrate for hyaluronidase, enzymatic degradation is not applicable as the primary reversal strategy.^10,11^ Available rheological evidence supports CMC-mediated obstruction and/or extrinsic compression as plausible drivers of acute VO, rather than PCL microsphere embolization, although further validation is required. Accordingly, acute management should prioritize reperfusion through dilution and mechanical dispersion rather than implant degradation.

The role of hyaluronidase in non-HA filler-induced VO warrants specific consideration. Although neither poly-L-lactic acid (PLLA), calcium hydroxylapatite (CaHA), nor PCL is a substrate for hyaluronidase, clinical observations suggest that the enzyme may nonetheless confer benefit in vascular events involving these fillers. Kadouch et al^12^ demonstrated rapid perfusion restoration on ultrasound when hyaluronidase was injected at perforator arterial roots in CaHA- and PLLA-induced occlusions, with two of three patients showing restored blood flow within minutes despite the absence of any direct degradative effect on the implanted material. The proposed mechanism is extravascular: enzymatic degradation of interstitial hyaluronan reduces perivascular tissue turgor and alleviates external vessel compression, while a parallel increase in interstitial permeability may improve the distribution of co-administered diluent.^13^

Because PCL-related VO events are rare and clinical outcome data are limited, protocol development currently relies on mechanistic reasoning, in vitro testing, and structured expert consensus.^14^ This manuscript presents a staged VO recognition and management algorithm for PCL-based fillers, integrating a targeted literature review, in vitro testing of PCL-CMC, and a modified Delphi consensus process. A practical graphic algorithm is provided to support rapid clinical decision-making in acute VO events.

## METHODS

### Targeted Literature Review

A targeted literature review was conducted using PubMed (January 2009–August 2025). Search terms combined concepts for PCL and CMC with vascular events in a filler context, limited to English-language publications. Key guidance documents, reviews, clinical series, and case reports were selected based on relevance to filler-induced VO, management strategies, or PCL physicochemistry. Reference lists were hand-searched, and consensus guidelines for HA and CaHA fillers were included to inform extrapolation where PCL-specific evidence was lacking. The full search strategy is provided in Appendix 1; a summary of key studies that informed Delphi item generation is presented in Supplemental Table 1. The review was intended to inform statement development rather than to constitute a formal systematic review; relevant background materials, including prior expert guidance and pharmacovigilance data, were summarized and circulated to panelists prior to Round 1.

### In Vitro Experimental Methods

Two experiments investigated the dilutional behavior of PCL-CMC. First, rheometry quantified viscosity at dilutions of 1:1, 1:2, and 1:5 (v/v) using a parallel-plate rheometer at a shear rate of 500 seconds^−1^, representative of small-vessel haemodynamics.^15^ Second, a flow test on inclined glass slides compared macroscopic flow characteristics between undiluted and diluted PCL-CMC, including time to homogeneous dispersion under manual mixing to simulate massage. Manual mixing was performed by rotating a spatula inside a Falcon tube containing the diluted product, using a gentle up-and-down motion until visibly homogeneous dispersion was achieved. This technique was selected to approximate the mechanical action of external manual massage; it represents gentle agitation rather than the turbulent mixing achievable with a vortex mixer, magnetic stirrer, or centrifuge. Both experiments were repeated using hyaluronidase solution (1500 IU/mL in saline) as the diluent to determine whether enzymatic activity conferred any additional benefit. Full experimental conditions and raw data are reported in Appendix 2.

### Expert Consensus: Modified Delphi Methodology

A modified Delphi consensus methodology^16^ was undertaken to develop a VO management algorithm specific to PCL-based fillers. A consensus approach was selected because of (1) the limited high-level evidence addressing VO management in PCL-based fillers, (2) the inapplicability of HA-derived hyaluronidase-centered guidance, and (3) recognized heterogeneity in real-world practice. Although guidance had previously been developed following the 2018 World Expert Meeting roundtable and was incorporated into manufacturer expert guidance materials (Sinclair Pharma Ltd., 2022), it had not been updated in 4 years. The present exercise was designed to update, expand, and formally validate prior recommendations in light of evolving clinical experience and emerging in vitro data. This consensus exercise was not prospectively registered.

The consensus panel comprised seven international key opinion leaders and certified PCL-CMC Expert Trainers: three plastic surgeons, three dermatologists, and one aesthetic physician, representing Portugal/UAE, Italy, Brazil, Romania, Egypt, Taiwan, and Mexico. Eligibility criteria included: (1) minimum 5 years’ experience injecting PCL-based fillers; (2) more than 500 PCL-CMC treatment sessions; and (3) direct clinical experience managing at least one filler-related adverse event. Panelists were identified by the study coordinators based on predefined criteria and invited via direct correspondence; all accepted. No public recruitment was undertaken. The coordinators facilitated the process, including development of draft materials and collation of responses, but did not participate in voting. No patients, carers, or members of the public were involved in the design or conduct of this exercise.

The process consisted of three rounds. In Round 1 (initial anonymous survey), panelists completed an anonymous online questionnaire (Microsoft Forms) evaluating statements on a 5-point Likert scale (Strongly Agree to Strongly Disagree). Free-text comments were permitted, and panelists could propose new items. Consensus was defined a priori as ≥80% of panelists selecting Strongly Agree or Agree. In Round 2 (face-to-face deliberation, June 2025), aggregated anonymized results and summary comments were presented to clarify divergent viewpoints and refine language; no voting occurred. Statements achieving consensus in Round 1 were not resubmitted unless substantively modified. In Round 3 (final anonymous survey), all revised or newly introduced statements were reassessed using the same Likert scale; statements achieving ≥80% agreement were retained. Data collection was conducted anonymously throughout. No pilot testing of the survey instrument was conducted. All surveys and meetings were conducted in English. The questionnaire is provided in Appendix 3.

As this work involved expert opinion synthesis without patient interaction or primary human subjects research, formal ethics committee approval was not required. All clinical images were used with written informed consent. The modified Delphi process is summarized in Figure 1.

**Figure 1.**
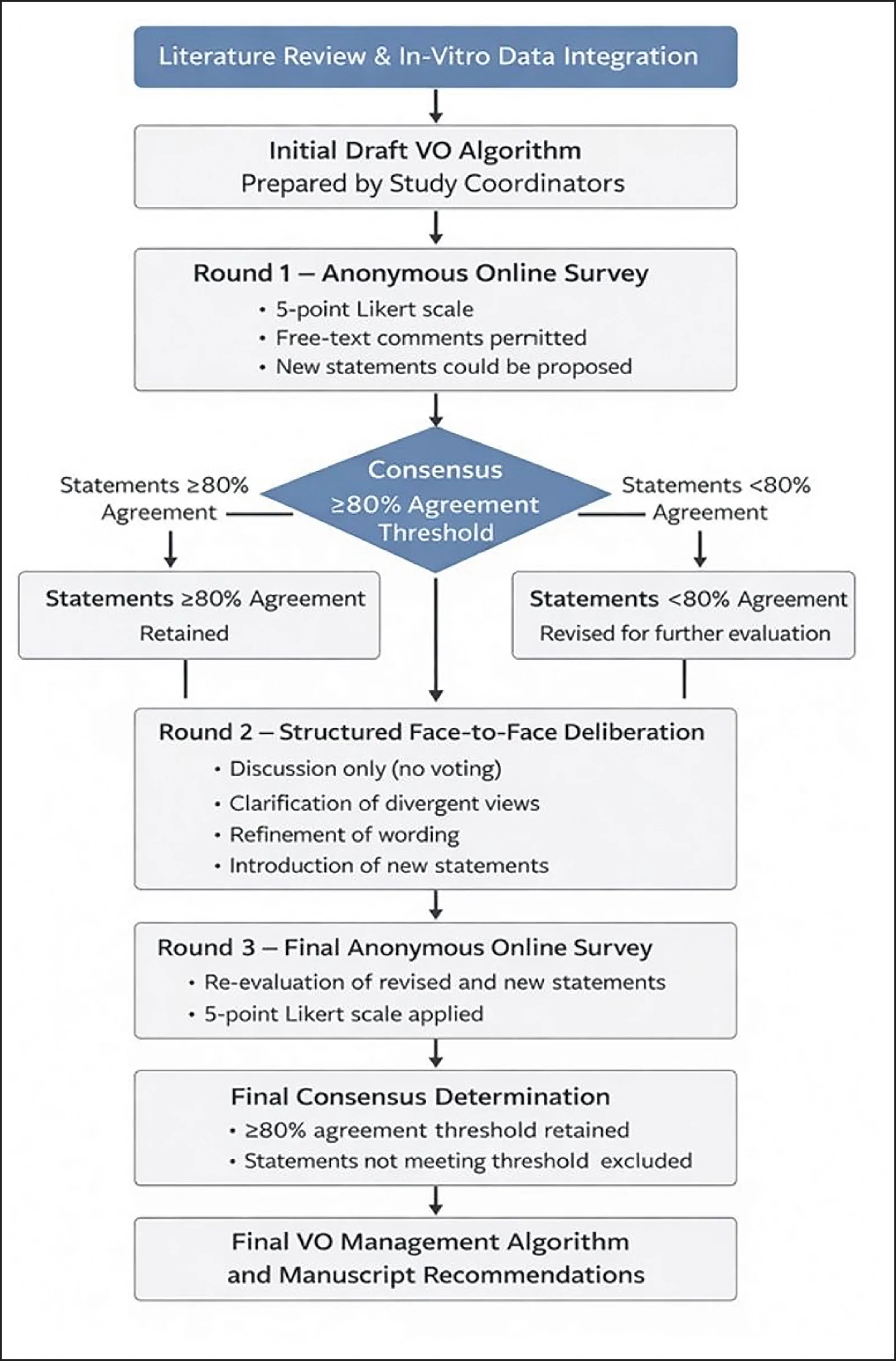
Modified Delphi process. Modified Delphi process for development of the PCL vascular occlusion management algorithm. An initial draft algorithm was generated from literature review and in vitro data. Three rounds were conducted (anonymous survey, structured discussion, and final anonymous survey), applying a predefined ≥80% agreement threshold for consensus. Statements meeting the threshold were retained; others were revised or excluded. The final output informed the management algorithm and manuscript recommendations. PCL, polycaprolactone.

## RESULTS

### Literature Review

No dedicated VO guideline for PCL-based fillers was identified. The published evidence is largely limited to clinical safety/efficacy studies, isolated VO case reports, and mechanistic studies. Two recent case reports describe successful management of PCL-induced VO,^17,18^ but management strategies were heterogeneous, highlighting the absence of a standardized protocol. Reported attempts at reperfusion commonly combine high-volume local infiltration or flushing with mechanical dispersion.

Established VO frameworks for HA^2,19^ and CaHA^20^ fillers provide useful principles for recognition, staging, and adjunctive treatments, but HA-centered algorithms emphasize enzymatic degradation with hyaluronidase as the primary intervention, which is not mechanistically applicable to PCL-CMC. A recent systematic review and meta-analysis of HA filler-induced VO (21 studies; 231 patients) reinforced the importance of early intervention, with improved outcomes when treatment is initiated within 6 hours, and reported overall resolution rates of 78.4% for skin necrosis and 45.2% for visual impairment.^21^ Adjunctive therapies including warmth, aspirin, corticosteroids, and vasodilators are mechanistically agnostic to filler type and were therefore incorporated as supportive elements within the PCL-specific protocol.^2,19^

Preclinical work further supports nonenzymatic strategies: in a rabbit model, combined heparin and nitroglycerin was associated with substantial reperfusion recovery and reduced thrombus formation compared with hyaluronidase alone in PCL embolism, suggesting that targeting secondary thrombus propagation and promoting flow restoration may improve outcomes.^22^ Taken together, the scarcity of PCL-specific outcome data and the mechanistic mismatch with HA-centered protocols necessitated investigation of the dilutional flushing and mechanical dispersion strategies that formed the basis of the in vitro experiments.

### In Vitro Results

#### Rheometry

Progressive dilution of PCL-CMC resulted in marked viscosity reductions (Table 1). At 1:5 dilution, viscosity decreased approximately 80-fold compared with the undiluted material, a magnitude that may be sufficient to materially reduce intraluminal flow resistance.

**Table 1.** Effect of Dilution on Ellansé Viscosity

Sample	Viscosity (Pa·s)^a^	Dilution factor	Viscosity reduction
Ellansé undiluted	3.40 ± 0.03	N/A	N/A
Ellansé 1:1 (v/v)	0.468 ± 0.002	2	7.3-fold
Ellansé 1:2 (v/v)	0.191 ± 0.001	3	17.8-fold
Ellansé 1:5 (v/v)	0.0415 ± 0.0008	6	81.9-fold
Water	0.001 ± 0.00004	N/A	N/A

^a^Mean ± range of triplicate measurements at ∼500 seconds−1, 20°C.

#### Flow Test

Undiluted PCL-CMC did not flow past the first quartile over 60 minutes. At 1:5 dilution, flow was immediate and identical to the water control. Progressive dilutions required increasing mixing times to achieve homogeneity: 60 seconds (1:1), 90 seconds (1:2), and 120 seconds (1:5) (Table 2).

**Table 2.** Time to Homogeneity With Manual Mixing

Sample	Dilution factor	Time to homogeneity (s)
Ellansé 1:1 (v/v)	2	60
Ellansé 1:2 (v/v)	3	90
Ellansé 1:5 (v/v)	6	120

When hyaluronidase solution was used as the diluent instead of water, no observable differences in flow behavior or mixing time were detected, indicating that enzymatic activity is not required for CMC gel dispersion.

### Expert Consensus Results

The consensus process was initiated in May 2025 and completed in November 2025. All seven invited panelists participated across all three rounds (response rate: 100%). In Round 1, 30 statements were evaluated as part of an initial Scientific Advisory Board questionnaire designed to assess current clinical practice, safety considerations, and unmet needs. Responses were used to identify areas of agreement, practice variation, and topics requiring further clarification.

During Round 2, findings were presented and discussed in a face-to-face meeting. Based on panel feedback, statements were refined, consolidated, and expanded, with particular focus on VO recognition and management. In Round 3, 16 statements relating specifically to VO management were formally assessed against predefined consensus criteria.

Final consensus was achieved on the majority of statements (Supplemental Table 2; Figure 2). The highest agreement was observed for mechanistic principles (CMC as primary contributor: full consensus) and core first-line management steps (immediate cessation, saline flushing, target 1:5 dilution: full consensus). A consensus based on majority of the experts (≥80%) was achieved for hyaluronidase retention, massage parameters, reassessment intervals, and second-line pharmacological measures. Lower agreement was noted for aspirin vs low molecular weight heparin (LMWH) prioritization (partial consensus, ≥70%), vasodilator selection (undetermined), and certain adjunctive measures. No deviations from the predefined methodology occurred.

**Figure 2.**
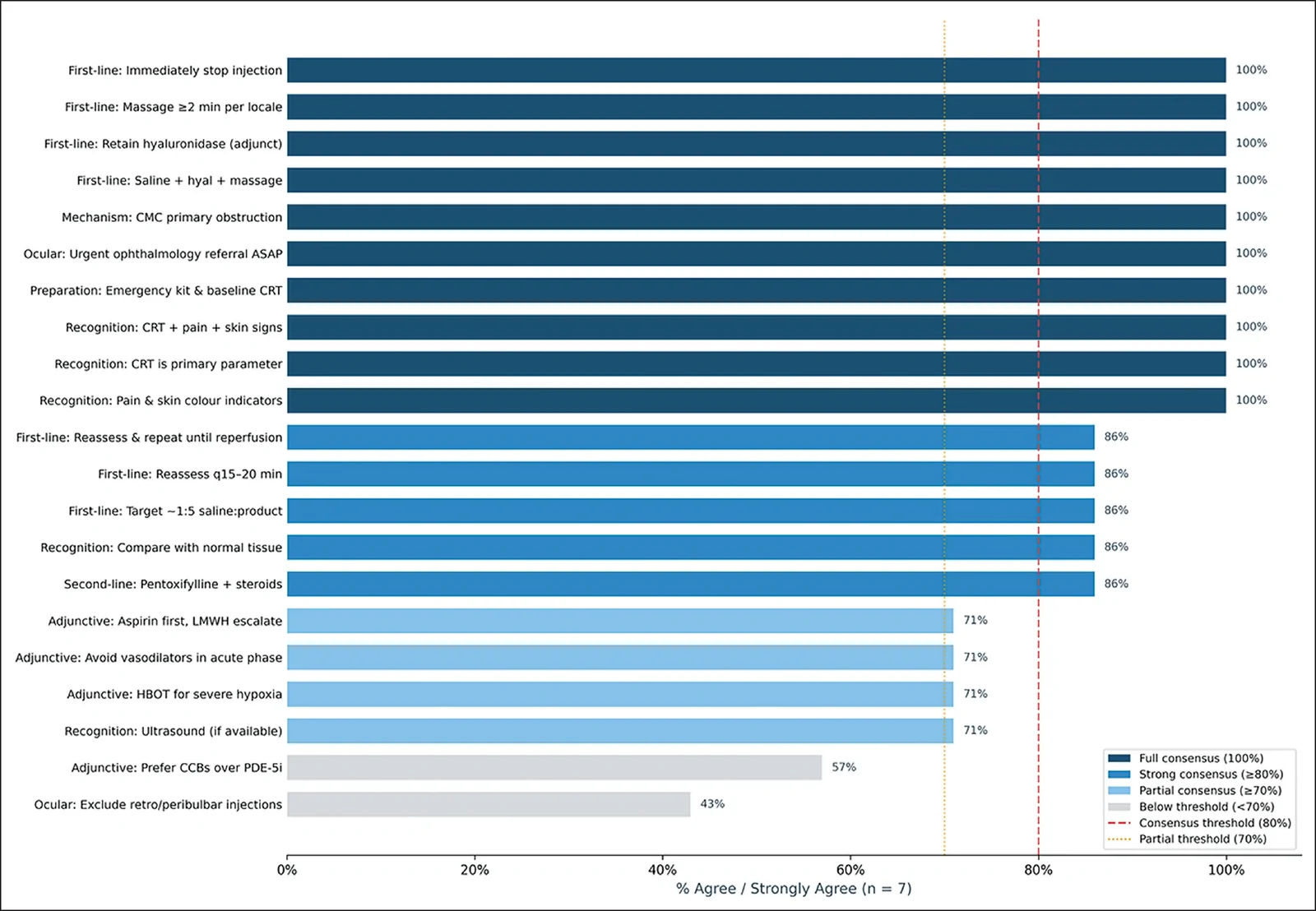
Modified Delphi results: agreement by protocol item. Agreement levels across Delphi statements for PCL-related vascular occlusion management. Bar graph showing the proportion of panelists selecting “Agree” or “Strongly Agree” for each protocol statement during the final Delphi round (*n* = 7). Full consensus corresponds to 100% agreement; strong consensus ≥80%; partial consensus ≥70%. The highest agreement was observed for mechanistic principles and core first-line management steps, whereas lower agreement was noted for selected adjunctive pharmacologic strategies and ocular procedural exclusions. The complete list of statements and consensus classifications is provided in Supplementary Material S4. PCL, polycaprolactone; CMC, carboxymethyl cellulose; CRT, Capillary refill time; HBOT, hyperbaric oxygen therapy; LMWH, low molecular weight heparin; CCB, calcium channel blockers.

### Management Protocol

Integrating in vitro findings, current literature, and expert consensus, a structured management protocol is proposed by the authors. The protocol addresses the distinct pathophysiology of PCL-based fillers, stratified by strength of evidence and consensus level. The finished algorithm is presented in Figure 3; an emergency kit summary is provided in Table 3.

**Figure 3.**
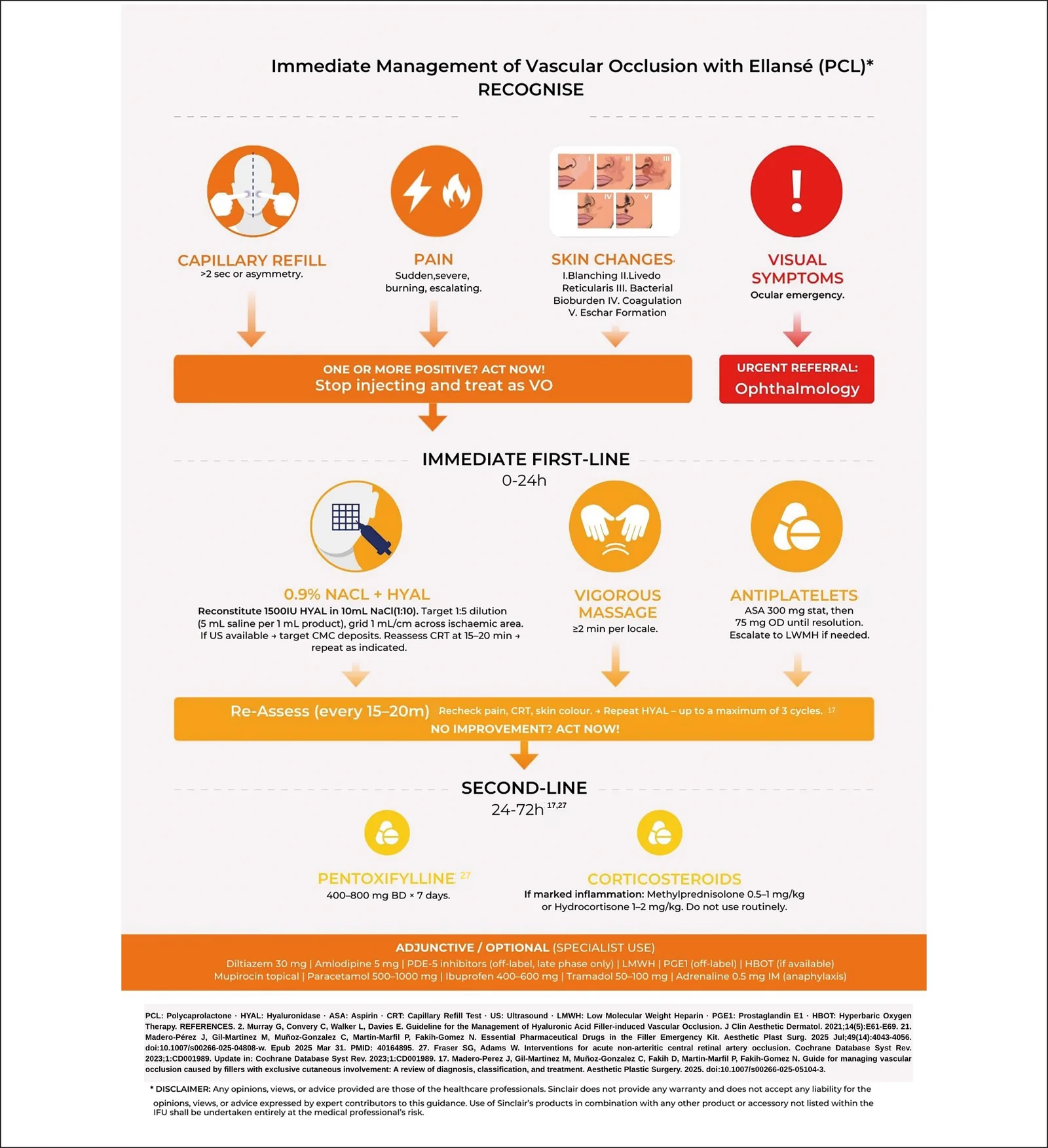
Immediate management of vascular occlusion with PCL-CMC (PCL)*. Staged management algorithm for vascular occlusion associated with polycaprolactone (PCL)-based fillers. The algorithm integrates in vitro evidence, literature review, and modified Delphi expert consensus. First-line management prioritizes high-volume saline flushing and mechanical dispersion, with hyaluronidase retained in a supportive role. Second-line and adjunctive therapies are stratified by evidence strength and consensus level. PCL, polycaprolactone; HYAL, hyaluronidase; ASA, aspirin; CRT, capillary refill test; US, ultrasound; LMWH, low molecular weight heparin; PGE1, prostaglandin E1; HBOT, hyperbaric oxygen therapy.

**Table 3.** Emergency Kit for Suspected PCL-Related Vascular Occlusion

Category	Medication	Dose/Specification
Core first-line	0.9% NaCl^a^	≥250 mL (target ∼1:5 dilution)
	Hyaluronidase (adjunctive)	1500 IU × 5 vials
Systemic first-line	Aspirin (or clopidogrel)	300 mg load → 75 mg daily
Escalation	LMWH^b^	Weight-adjusted dosing
Second-line (24-72 h)	Pentoxifylline	400-800 mg BD^c^
	Methylprednisolone	0.5-1 mg/kg (if marked inflammation)
Adjunctive	CCBs^d^ (diltiazem/amlodipine)	After CMC^e^ dispersal only
	HBOT^f^	If available
Supportive	Analgesia, topical mupirocin	Standard dosing

^a^Sodium chloride

^b^Low-molecular-weight heparin

^c^Bis in die, twice daily

^d^Calcium channel blockers

^e^Hyperbaric oxygen therapy

^f^Carboxymethyl cellulose

#### Recognition and Continuous Assessment

Capillary refill time (CRT) is the primary diagnostic and monitoring parameter. A CRT exceeding 2 seconds, or a difference of more than 2 seconds compared with adjacent unaffected tissue, suggests arterial compromise. A baseline CRT should be recorded prior to treatment. During suspected VO, CRT should be reassessed at 15-20 minute intervals. Additional indicators include sudden escalating pain and skin color changes, classified into five stages from blanching to necrosis (Figure 4).^2^ For injections in high-risk vascular territories (nose, glabella, and forehead), a 30-minute postinjection observation period is recommended.^19^ Where available, ultrasound may assist in product localization and guide targeted intervention.^23,24^ If any visual symptoms are reported, immediate referral to ophthalmology or a stroke unit should be initiated, as irreversible retinal damage can occur within 15 minutes of occlusion.^25^

**Figure 4.**
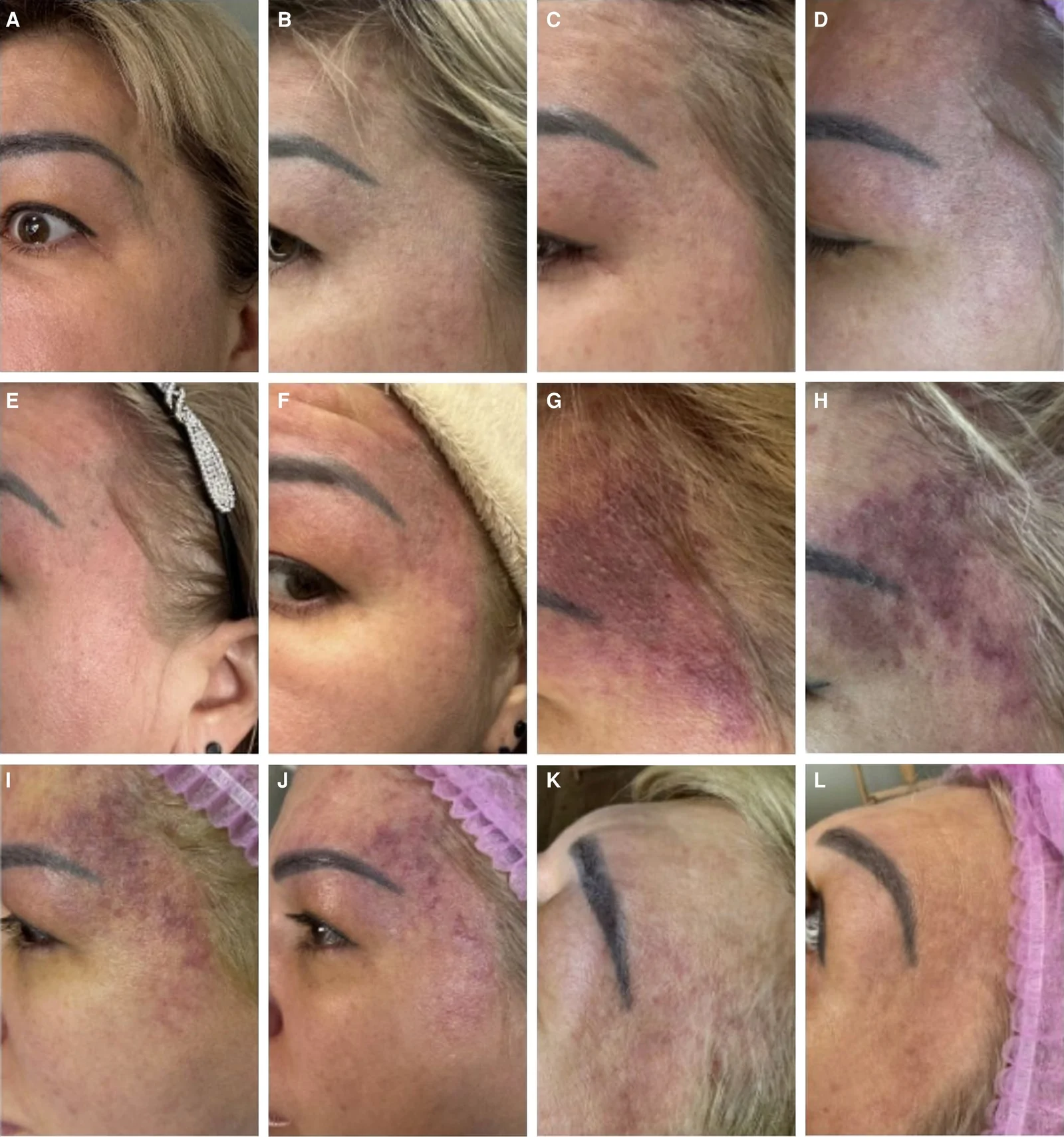
Staged clinical evolution and successful reversal of vascular occlusion following PCL-CMC injection in the temporal region. Temporal evolution of cutaneous vascular compromise following PCL-CMC injection and staged clinical recovery under multimodal management in a 42-year-old female patient. Representative clinical images demonstrate sequential progression: (A, B) Stage I, arterial compression, acute blanching; (C, D) Stage II, hypoperfusion, livedo reticularis at 24-48 hours; (E, F) Stage III, inflammation, early inflammatory compromise (Days 3, 4); (G, H) Stage IV, ischemia/hemorrhage, established ischemic changes (Days 4, 5); (I, J) Stage V, reperfusion and early remodeling (Days 7, 8); (K, L) Stage VI, resolution by Day 30. Images courtesy of Malovana A., used with patient consent.

#### First-Line Management (0-24 Hours)

Injection must be stopped immediately upon suspicion of VO. First-line management consists of high-volume isotonic saline infiltration combined with immediate vigorous mechanical massage. Hyaluronidase may be co-administered as an adjunctive agent. The target dilutional volume is approximately 5 mL of saline per 1 mL of product (≈1:5 dilution), informed by the in vitro viscosity data. Dilutional volume is delivered in a grid pattern (1 mL per 1 cm across the ischemic area), injected gently with the aim of saturating the affected tissue. Massage should follow each infiltration cycle for a minimum of 2 minutes per affected locale, with extension permitted based on clinical response.

CRT should be reassessed at 15-20 minute intervals during the active infiltration phase. Flushing and massage cycles are repeated while each successive cycle continues to produce measurable improvement in CRT, capillary perfusion, or pain. The active infiltration phase is concluded when CRT returns to baseline or two consecutive cycles produce no further improvement; management transitions to the adjunctive pharmacological measures and postacute care described below.

If ultrasound is available, targeted injection of visible CMC deposits may be considered; however, while this approach is described for HA-based VO, its efficacy in PCL-based fillers has not yet been established.^26^

Concurrently, aspirin is given at a loading dose of 300 mg, continued daily at 75 mg until resolution.^27^ In cases of extensive ischemia, delayed presentation, or inadequate response, escalation to LMWH may be considered following appropriate contraindication screening.

#### Second-Line and Adjunctive Management (24-72 Hours)

Where escalation is needed, pentoxifylline (400-800 mg twice daily for 1 week) decreases blood viscosity and improves microvascular perfusion.^28^ Corticosteroids (methylprednisolone 0.5-1 mg/kg) can be administered for marked inflammation but should not be used routinely due to infection risk and impaired wound healing.^2^ Vasodilators are avoided in the acute phase because of the risk of distal embolic propagation; after CMC gel dispersal, calcium channel blockers may support tissue reperfusion. Additional measures include hyperbaric oxygen therapy (HBOT) if available,^29^ topical mupirocin, and multimodal analgesia.^19^

#### Postacute Management (Day 3 and Beyond)

Beyond the initial 72-hour management window, ongoing assessment and wound care remain essential until complete tissue resolution. From Day 3 onwards, clinical review should assess for signs of progressive ischemia, evolving necrosis, or secondary infection. If tissue viability remains uncertain, repeat saline flushing and massage may be attempted if residual product is suspected on clinical grounds or ultrasound. Once reperfusion is confirmed, antiplatelet therapy (aspirin 75 mg daily) should be continued until full tissue resolution. Wound management including topical antibiotics (eg, mupirocin), appropriate dressings, and multimodal analgesia should be maintained as clinically indicated.^30^ Any patient with ongoing or worsening signs should be referred promptly to a vascular specialist, plastic surgeon, or wound care service. Late sequelae including hyperpigmentation, scarring, or volume loss may require management after tissue stabilization; these fall outside the scope of this acute protocol and are addressed in dedicated references.^31^

#### Preinjection Safety and Documentation

Prophylactic measures include preparation of an emergency kit, baseline bilateral CRT measurement, and pre-established escalation plans and referral pathways. Cannula use is strongly supported over needles, with one review reporting a 77.1% reduction in VO risk with microcannulas compared to needles (1 per 40,882 vs 1 per 6410 syringes, respectively).^32^ Slow, low-pressure injection in small increments (≤0.1-0.2 mL) is recommended.^2^ Safe injection techniques and recommended anatomical planes of injection for each treatment zone (including the nose, forehead, temples, and infraorbital region) should be respected; detailed injection technique guidance is beyond the scope of this protocol and is addressed in dedicated anatomical references.^33^ All incidents are documented with written notes and photographic records, and all complications reported to the product manufacturer through standardized channels.

## DISCUSSION

### Mechanistic Rationale

Two mechanisms may plausibly contribute to acute PCL-associated VO: mechanical obstruction by the CMC carrier gel, whose undiluted viscosity is sufficient to occlude small vessels; and embolization by PCL microspheres (25-50 µm), which are of a caliber capable of lodging within distal arterioles, particularly following inadvertent intra-arterial injection. Three lines of evidence favor CMC-mediated obstruction as the dominant mechanism in most acute events: the microspheres are smooth, spherical, and narrowly size-distributed, reducing the likelihood of aggregation;^6,7^ the in vitro data presented here show that the CMC component alone provides sufficient flow resistance to occlude small vessels; and the clinical signature—sudden pain and blanching within seconds of injection— is consistent with gel-mediated rather than particulate obstruction.^17,18^

A temporal argument further supports this interpretation. PCL microspheres persist with minimal mass loss for many months—up to approximately 18 months for Ellansé S and 24 months for Ellansé M.^7,12^ If the microspheres themselves were the principal driver of occlusion, a sustained or delayed vascular risk across this lifespan would be expected; no such delayed events have been reported in pharmacovigilance or published clinical series,^8,14^ supporting a transient, gel-mediated mechanism. This mechanistic distinction underpins the departure from HA-centered algorithms.

Distinguishing the two mechanisms has therapeutic implications. CMC-mediated obstruction is directly amenable to the dilution-and-massage approach proposed here. Microsphere embolization, once established, cannot be expected to respond to dilution or external massage; in such cases the protocol's value derives from its adjunctive components—antiplatelet or anticoagulant therapy to limit secondary thrombus propagation, and supportive measures to maximize collateral perfusion. The protocol therefore provides a degree of coverage if microsphere embolization is the dominant component in a given event. We acknowledge that the relative contribution of these two mechanisms remains unconfirmed in vivo, and that further mechanistic research is required to establish which is the principal driver of acute PCL-associated VO.

### Primary Intervention: Saline Dilution and Massage

High-volume saline dilution (5 mL per 1 mL product). High-volume saline flushing is the primary therapeutic intervention. The recommendation of 5 mL saline per 1 mL product derives directly from the in vitro viscosity measurements. The 1:1 dilution reduced viscosity approximately 7-fold, 1:2 approximately 18-fold, and 1:5 approximately 80-fold, bringing the CMC carrier to near-aqueous levels with immediate unrestricted flow on slide testing. At the 1:5 dilution level, the CMC carrier should lose the viscosity required to maintain sufficient resistance to occlude an artery.

Massage duration: minimum 2 minutes per cycle. The in vitro flow experiments determined that achieving homogeneous mixing required progressively longer times: 60 seconds for 1:1, 90 seconds for 1:2, and 120 seconds for 1:5 dilution. In vivo conditions—including tissue resistance, non-uniform force application, and variable saline distribution—likely require longer than bench stirring to achieve comparable results. Practitioners are explicitly encouraged to extend massage beyond 2 minutes and apply more vigorous mechanical manipulation when clinically safe.

Hyaluronidase: rationale for retention. The decision to retain hyaluronidase despite its inability to degrade CMC or PCL was based on multiple considerations. Kadouch et al's ultrasound-guided study of CaHA- and PLLA-induced VOs (neither degradable by hyaluronidase) demonstrated rapid perfusion restoration when hyaluronidase was injected at perforator arterial roots; two of three patients showed restored blood flow on ultrasound within minutes.^13^ Although the exact mechanism is unclear, extravascular hyaluronidase may reduce perivascular tissue turgor by degrading extracellular hyaluronan, potentially alleviating external vessel compression. Furthermore, hyaluronidase increases interstitial permeability,^34^ which may improve distribution of the saline diluent. The protocol explicitly reframes hyaluronidase from a primary treatment to a supportive role, acknowledging the mechanistic differences between filler types.

### Diagnostics and Reassessment Rationale

Of the three diagnostic signs—CRT, pain, and skin changes—CRT was selected as the principal parameter due to its objectivity and responsiveness to changes in tissue perfusion. The experts noted, however, that in practice, sudden, severe, or escalating pain often represents the earliest clinical sign; unexpected pain within seconds of injection should immediately trigger cessation and tissue assessment. The reassessment interval of 15-20 minutes aligns with CRT kinetics data showing that perfusion changes become detectable 6-8 minutes after fluid expansion, with maximal improvement at 10-12 minutes.^35^ This window provides sufficient time for saline diffusion and massage-mediated dispersion before evaluating therapeutic response.

### Areas of Disagreement

#### Aspirin vs Low-Molecular-Weight Heparin

The HA-derived literature supports aspirin as first-line antiplatelet therapy; however, two of seven panelists strongly favored LMWH as initial strategy. In a comparative rat epigastric flap model, intra-arterial PCL produced substantially fewer lumen-filling thrombi than HA, despite comparable endothelial injury, supporting the interpretation that the thrombotic burden may be less dominant in PCL events—making platelet inhibition a reasonable and proportionate first-line approach.^36^ Limitations include the animal model and large-vessel context, which may not fully replicate facial microvascular dynamics. Conversely, Khan et al's rabbit auricular artery model showed heparin plus nitroglycerin achieved ∼80% relative perfusion recovery in PCL embolism by day 7, attenuating thrombosis and inflammatory cell aggregation.^22^ However, the observed benefit reflects a combination therapy, not anticoagulation alone. The protocol therefore presents both as acceptable: aspirin as default (accessibility, ease of administration, HA-guideline precedent) and LMWH as an equally valid alternative for extensive ischemia, delayed presentation, or poor response to initial mechanical measures.

#### Vasodilator Choice

The literature supports calcium channel blockers (amlodipine, diltiazem) as the preferred vasodilatory option,^19^ while the expert panel expressed preference for sildenafil (PDE-5 inhibitor). Comparative data are lacking; further research is needed.

#### Nitroglycerin Exclusion

Despite positive preclinical data from Khan et al,^22^ nitroglycerin was excluded from the routine protocol for three reasons. First, conflicting preclinical evidence: Hwang et al's 2016 rabbit ear study found no statistically significant improvement in perfusion with topical nitroglycerin across four filler types including PCL, and nitroglycerin-treated ears appeared more congested than controls, suggesting potential harm through embolic propagation.^37^ Second, established guideline recommendations oppose routine use: the 2021 CMAC guideline^2^ and the 2025 Madero-Pérez review^19^ both advise against acute-phase nitroglycerin given the lack of supporting evidence and the risk of venous congestion. Third, practical constraints including contraindications (concurrent PDE-5 inhibitors, severe aortic stenosis), hemodynamic adverse effects (hypotension, syncope), and the requirement for continuous cardiovascular monitoring that is impractical in community aesthetic settings.^38,39^ Nitroglycerin remains a potential second-line option for specialist-supervised, refractory cases with proven intra-arterial occlusion and inadequate response to mechanical dilution plus LMWH—positioned as explicitly off-label, requiring informed consent and hemodynamic monitoring.

#### Ophthalmological Management

No published cases of permanent visual loss attributed to PCL-CMC exist; nevertheless, the panel achieved full consensus that any visual symptom—transient blur, scotoma, ptosis, painful eye movement, or frank vision loss—warrants immediate referral to ophthalmology or to a stroke service capable of acute neurovascular intervention. While transfer is being arranged, the injecting clinician may initiate intermittent digital ocular massage^39^ and carbon dioxide rebreathing to promote retinal arterial vasodilation;^40,41^ these measures are low-risk and should not delay transfer. Specialist interventions that may be considered at the receiving center—including intra-arterial thrombolysis, HBOT,^42^ and anterior chamber paracentesis—lie outside the scope of clinic-based aesthetic practice; guidance on these interventions, including patient-selection criteria and therapeutic windows, is available in dedicated consensus documents on filler-related visual compromise^27,43-45^ and on the management of central retinal artery occlusion more broadly.

### Consensus on Fundamentals

The achievement of full consensus on foundational principles—CMC-mediated obstruction, immediate cessation, saline dilution targeting 1:5, and urgent ophthalmological referral for visual symptoms—reflects the strength of in vitro and preclinical evidence supporting gel-mediated mechanical obstruction as the dominant mechanism. This consolidation of mechanistic principles contrasts productively with the disagreement on adjunctive choices (aspirin vs LMWH, CCB vs PDE-5i), which appropriately acknowledges that the primary intervention can be well-supported while secondary therapies remain empirically uncertain. The protocol is designed to provide a clear default pathway for emergency management while preserving clinical flexibility for individual patient circumstances.

### Strengths and Limitations

This work integrates mechanistic evidence, in vitro testing, and structured expert consensus to address a clinically important but infrequent complication. The achievement of full consensus on foundational principles reflects the strength of the in vitro and preclinical evidence base. The ACCORD reporting guideline was followed to ensure transparent documentation of the consensus process.^46^

Several limitations should be considered. The in vitro experiments were conducted under controlled conditions using a limited number of product batches and do not replicate in vivo tissue architecture, vascular dynamics, or temperature variation. Specifically, bench mixing cannot fully replicate clinical massage. External massage in clinical practice is applied through soft tissue with variable force, depth and tissue resistance, and the mechanical energy transferred in vivo cannot be assumed to be equivalent to the bench-top protocol. The flow test should therefore be interpreted as a directional demonstration of dispersion behavior under gentle mixing, rather than a quantitative simulation of what external massage achieves clinically. The protocol is therefore evidence-informed rather than prospectively validated. Some variability remained in adjunctive therapy preferences, reflecting limited comparative evidence. The panel comprised seven experts who are paid consultants for the product manufacturer; although the consensus process was conducted with anonymized voting and the coordinators held no voting rights, this relationship may introduce perception of bias. The rarity of PCL-related VO, while clinically reassuring, restricts availability of outcome data and necessitates reliance on mechanistic reasoning and clinical experience. The nonanonymized face-to-face Round 2 discussion may have influenced subsequent voting, although anonymity was maintained in both survey rounds.

Direct in vivo evidence—animal histology or human biopsy—confirming the primacy of CMC-mediated obstruction over PCL microsphere embolization is currently absent. The relative contribution of these two mechanisms in any given event cannot be quantified from the present data, and residual particulate obstruction cannot be excluded, particularly following inadvertent intra-arterial injection.

### Future Research Priorities

Prospective multicenter data are required to validate the saline and massage parameters proposed. In vivo perfusion and histological models should clarify saline dispersion dynamics, the role of hyaluronidase in PCL-related occlusion, and the relative contribution of CMC-mediated obstruction vs PCL microsphere embolization. Comparative trials of antiplatelet, anticoagulant, and vasodilator strategies are needed to resolve therapeutic uncertainty.

## CONCLUSIONS

VO with PCL-based fillers requires a mechanistically distinct management approach from HA-related events. The CMC carrier gel—rather than the PCL microspheres—may be the primary contributor to acute obstruction, and unlike HA fillers, it cannot be degraded enzymatically. This consensus-derived protocol therefore prioritizes high-volume saline dilution (target 1:5 ratio) combined with sustained mechanical massage as first-line therapy, with hyaluronidase retained in a supportive rather than primary role. Prevention remains paramount:anatomical expertise, cannula use, low-pressure microaliquot delivery, and preparedness with a stocked emergency kit represent the most effective strategy against VO. While the in vitro evidence and expert consensus provide a structured, evidence-informed foundation for clinical decision-making, these recommendations require prospective multicenter validation to confirm optimal dosing parameters and refine adjunctive therapy selection.

## Supplemental Material

This article contains supplemental material located online at https://doi.org/10.1093/asjof/ojag167.

## Supplementary Material

ojag167_Supplementary_Data
