# Supplementary material for: Staged Management of Vascular Occlusion With Ellansé (Polycaprolactone): A Modified Delphi Expert Consensus
Source: Aesthet Surg J Open Forum. 2026 Sep 30;8:ojag167. doi: 10.1093/asjof/ojag167 (PMC13625641; doi:10.1093/asjof/ojag167)
Supplement: Supplementary file 1 [file ojag167_supplementary_data.zip › Appendix 1.docx]

**Supplementary Material S1: Search Strategy**

A targeted literature review was conducted using PubMed from January 2009 to August 2025. The following search strategy was employed:

*((polycaprolactone OR PCL OR PCL-based OR PCL microsphere$ OR ellanse OR sculptra OR lanluma OR biostimulator$ OR carboxymethylcellulose OR CMC carrier OR CMC gel).ti,ab,kf.) AND ((vascular adj3 (occlusion$ OR complication$ OR compromise$)) OR (arterial adj3 (occlusion$ OR embolism OR embolization)) OR ischem$ OR necros$ OR infarct$ OR vascular event$).ti,ab,kf. AND (dermal filler$ OR soft tissue filler$ OR injectable filler$ OR facial filler$).ti,ab,kf.*

Articles were limited to English-language publications. Key guidance documents, reviews, clinical series, and case reports were selected based on relevance to filler-induced VO, management strategies, or PCL physicochemistry. Reference lists of eligible articles were hand-searched to identify additional sources. Consensus guidelines for HA and CaHA fillers were included where PCL-specific evidence was lacking.
