# Supplementary material for: Staged Management of Vascular Occlusion With Ellansé (Polycaprolactone): A Modified Delphi Expert Consensus
Source: Aesthet Surg J Open Forum. 2026 Sep 30;8:ojag167. doi: 10.1093/asjof/ojag167 (PMC13625641; doi:10.1093/asjof/ojag167)
Supplement: Supplementary file 1 [file ojag167_supplementary_data.zip › Appendix 2.docx]

**APPENDIX 2: In-Vitro Experimental Protocols**

***Viscosity Characterisation (Rheometry)***

The viscosity of undiluted Ellansé® and samples diluted with water at ratios of 1:1, 1:2, and 1:5 (v/v) was characterised using a TA Instruments DHR-2 rheometer with a 32 mm parallel-plate geometry and a 500 µm gap. A flow sweep test was performed across a shear rate range of 10–1000 s⁻¹. Viscosity values were recorded at a shear rate of 500 s⁻¹, which lies within the physiological microvascular range (10²–10³ s⁻¹) reported for small arteries and arterioles.^10^ Water served as the control. The rheometer was calibrated annually per manufacturer specifications. Each sample was measured in triplicate at room temperature.

***Dilutional Flow Test***

Approximately 0.3 mL of each sample was deposited at the top edge of clean glass microscope slides marked with four quartiles. Water served as the control. Slides were positioned at a 45° angle and left undisturbed at room temperature. The extent of downward flow was measured at 15-minute intervals over one hour by recording the quartile reached by the leading edge. Additionally, the time required for dilutions to reach homogeneous dispersion through manual mixing with a spatula (simulating clinical massage) was determined. Homogeneity was defined as the absence of visible product upon inspection. Of note, manual mixing was performed by rotating a spatula inside a Falcon tube containing the diluted product, using a gentle up-and-down motion to combine the materials. This was selected as the closest available bench-top approximation of external manual massage; it is a gentle agitation and does not reach the turbulent mixing achievable by vortex, magnetic stirring, or centrifugation.

***Hyaluronidase Comparison***

To determine whether hyaluronidase conferred any advantage over water during dilution or mixing, the flow and mixing experiments were repeated using hyaluronidase reconstituted in saline (1500 IU in 10 mL) as the diluent instead of water, at the same volumetric ratios and conditions.
