# Supplementary material for: Staged Management of Vascular Occlusion With Ellansé (Polycaprolactone): A Modified Delphi Expert Consensus
Source: Aesthet Surg J Open Forum. 2026 Sep 30;8:ojag167. doi: 10.1093/asjof/ojag167 (PMC13625641; doi:10.1093/asjof/ojag167)
Supplement: Supplementary file 1 [file ojag167_supplementary_data.zip › Appendix 3.docx]

**appendix 3: QUESTIONNAIRES**

The following questionnaires were administered electronically via Microsoft Forms. In both rounds, panellists rated each statement on a 5-point Likert scale (Strongly Agree to Strongly Disagree) unless otherwise indicated. Free-text comments were permitted throughout. Consensus was defined a priori as ≥80% of panellists selecting Strongly Agree or Agree.

**Round 1 Questionnaire: Ellansé® Scientific Advisory Board Survey**

*Administered: May 2025 (Istanbul). This initial survey assessed current clinical practice, product perceptions, safety considerations, and unmet needs to inform statement development for the modified Delphi consensus. Twenty-four questions were administered; questions 9–19 (relating to adverse event management and clinical protocols) directly informed the development of VO-specific consensus statements in subsequent rounds.*

**Q9.** What do you believe is the most commonly encountered adverse event associated with Ellansé® in clinical practice? *[Non-inflammatory nodules / Inflammatory nodules / Granulomas / Overfilling or contour irregularities / Vascular occlusion / Persistent swelling or oedema / Induration or firmness at the injection site / Other]*

**Q10.** In your experience, what has been the most common cause of Ellansé® adverse events? *[Overfilling / Dilution of Ellansé® / Improper placement or incorrect layer / Poor patient selection / Product related / Other]*

**Q11.** How confident are you in the current clinical guidance and protocols currently available for managing vascular occlusion with Ellansé®? *[Strongly Agree / Agree / Neutral / Disagree / Strongly Disagree / Other]*

**Q12.** Do you agree that this should be the approach when clinically assessing a delayed onset nodule associated with Ellansé®? [Algorithm presented] *[Yes / No / Other]*

**Q13.** How confident are you in the clinical guidance and protocols currently available for managing non-inflammatory nodules with Ellansé®? *[Extremely confident / Strongly Agree / Neutral / Somewhat confident / Extremely not confident / Other]*

**Q14.** Do you agree that for management of single/multiple inflammatory lesions (pathology unclear) post Ellansé® treatment, the best approach is: Step 1: If fluctuant, drain with swab for extended culture; consider bloods (CRP, ESR, FBC). Step 2: If moderate–severe inflammation, consider punch biopsy for histopathology or extended culture (ideally prior to antibiotics). Step 3: Broad-spectrum antibiotics: 1st line: clarithromycin 500 mg BD PO × 14 days + doxycycline 100 mg BD PO × 14 days; 2nd line: ciprofloxacin. *[Yes / No / Yes but I have something to add / Other]*

**Q15.** How often do you use high-frequency ultrasound in your practice when managing adverse events? *[Always / Often / Sometimes / Rarely / Never]*

**Q16.** If you do not use high-frequency ultrasound, would you consider using it in management of your adverse events? *[Yes / No / Maybe / N/A: I do use it]*

**Q17.** Have you used collagenase before in managing non-inflammatory nodules caused by Ellansé®? *[Yes / No, haven’t had the chance to / No, doesn’t work]*

**Q18.** Do you use any additional or personal protocols, beyond standard guidance, for managing adverse events related to Ellansé®? (e.g., radiofrequency, allopurinol, hyaluronidase, etc.) *[Yes, frequently / Occasionally / Rarely / Never]*

**Q19.** If yes, please briefly describe your approach. *[Free text]*

*Note: Questions 1–8 and 20–24 addressed product positioning, training, and stacking protocols; these informed broader advisory board recommendations but are not directly related to the VO consensus and are therefore not reproduced here.*

**Round 2: Face-to-Face Deliberation Material**

*Circulated and discussed: June 2025. Following Round 1, aggregated anonymised results and initial in-vitro findings were presented to the panel. The following topics were discussed, with open questions posed to the panel. Panellist feedback was used to refine statements for Round 3. Key discussion points and panel feedback are summarised below.*

**Topic 1: Diagnostic Indicators and Monitoring**

Panellists were presented with the proposed diagnostic triad: (1) capillary refill time (CRT), (2) pain, and (3) skin colour changes. The panel was asked to rank their importance and comment on monitoring frequency. Key feedback included the recommendation that CRT should be positioned as the most critical parameter, with comparison to adjacent normal tissue as a mandatory assessment step. Multiple panellists supported the inclusion of PDE-5 inhibitors (sildenafil, tadalafil) as late-phase vasodilatory options, while noting limited evidence.

**Topic 2: Vasodilators and PDE-5 Inhibitors**

In-vitro and literature evidence was presented showing that acute-phase vasodilators carry a risk of worsening ischaemia through distal embolic propagation. Published reviews advise against early vasodilator use but support cautious post-reperfusion application. Among vasodilatory agents, calcium channel blockers were identified as having the most favourable evidence and safety profile. PDE-5 inhibitors were discussed as theoretical late-phase adjuncts with limited clinical evidence; concerns included reports of retinal vascular occlusion following sildenafil use. The panel was asked whether PDE-5 inhibitors should be listed as optional late-phase adjuncts only, with CCBs as preferred. No formal vote was taken; this was resolved in Round 3.

**Topic 3: Aspirin vs LMWH**

One panellist presented a strong case for LMWH as the preferred first-line anticoagulant/antiplatelet agent, citing faster onset and evidence of microcirculatory benefit in preclinical PCL embolism models. However, direct comparative evidence between LMWH and aspirin in filler-associated VO was acknowledged as absent. The proposed solution was to maintain aspirin as the accessible first-line default and position LMWH as an escalation option. This was formally voted upon in Round 3.

**Topic 4: CMC Dispersibility and Flushing Protocol**

In-vitro rheometry and flow test results were presented to the panel, demonstrating that Ellansé® viscosity drops sharply with progressive dilution (approximately 80-fold at 1:5 at physiological shear rates), that the mixture reaches homogeneity after approximately 90 seconds of manual mixing, and that hyaluronidase confers no measurable benefit over saline alone for CMC dispersion. The panel was asked whether the 1:5 saline-to-product ratio was a realistic and defensible recommendation, whether hyaluronidase should be retained or omitted, and whether high-volume saline flushing with massage should be positioned as the evidence-based first-line management. Key panellist feedback endorsed the saline flushing approach, supported retention of hyaluronidase for its tissue permeability effects (despite no direct CMC effect), and emphasised that in-vivo dispersion likely requires higher volumes and more vigorous manipulation than bench conditions.

**Topic 5: Ocular Complications and Referral Timing**

The panel discussed evidence showing irreversible retinal ischaemic injury can occur within 15–20 minutes of occlusion, making the previously proposed 90-minute referral window inadequate. One panellist noted that ophthalmology review within 30 minutes is impractical in most real-world settings and that ophthalmologists rarely intervene directly (retrobulbar injections being of uncertain efficacy). The consensus was to recommend referral ‘as soon as possible’ while noting the critical window. CO₂ rebreathing as a supportive measure to induce retinal vasodilation was discussed and positioned as an optional adjunct.

**Topic 6: Massage and Mechanical Disruption**

Panellists emphasised that saline should be injected slowly without forceful flushing to avoid worsening compression, and that vigorous mechanical massage is essential for gel redistribution. The proposed 5 mL saline per 1 mL product ratio was endorsed as proportionate to expected occlusion volumes (typically 0.1–0.2 mL of product). Continuous CRT monitoring was confirmed as the most reliable real-time indicator of therapeutic response.

**Round 3 Questionnaire: Ellansé® Vascular Occlusion Management**

*Administered: November 2025. This final survey formally assessed statements relating specifically to VO recognition and management following refinement in Round 2. Statements achieving ≥80% agreement (Strongly Agree or Agree) were retained as consensus recommendations.*

**Q1.** In suspected Ellansé® VO: (1) CRT (most critical), (2) pain, and (3) skin colour/changes should be considered diagnostic indicators. *[Strongly Agree / Agree / Neutral / Disagree / Strongly Disagree / Other]*

**Q2.** Rank the most important steps after a VO by Ellansé® (most important to least important): *[Ranking: Test CRT >2 sec or asymmetry / Compare perfusion to normal tissues / Pain (sudden, severe, burning, escalating) / Skin changes]*

**Q3.** Comparing perfusion in the affected area with adjacent normal tissue helps assess improvement over time and should be included as a mandatory step in the algorithm. *[Strongly Agree / Agree / Neutral / Disagree / Strongly Disagree / Other]*

**Q4.** Although we know that hyaluronidase has no effect on CMC or PCL microspheres, it should be included in the protocol as it increases tissue permeability to increase the chances of saline reaching the occlusion. *[Strongly Agree / Agree / Neutral / Disagree / Strongly Disagree]*

**Q5.** First-line management of VO (0–24 hrs): Isotonic saline flushing (NaCl 0.9%) + hyaluronidase (accessory agent) + immediate massage is an appropriate first-line management step for suspected Ellansé® VO. *[Strongly Agree / Agree / Neutral / Disagree / Strongly Disagree / Other]*

**Q6.** Second-line management (24–72 hours): Pentoxifylline 400–800 mg BD × 7 days and corticosteroids (methylprednisolone 0.5–1 mg/kg or hydrocortisone 1–2 mg/kg). *[Strongly Agree / Agree / Neutral / Disagree / Strongly Disagree]*

**Q7.** Third-line (adjunctive/optional, 24–72 hrs): Do you agree with the following? Avoid acute vasodilators; if used after reperfusion, prefer CCBs (diltiazem 30 mg / amlodipine 5 mg); do not combine vasodilators with PDE-5 inhibitors. PDE-5 inhibitors (off-label, late phase only): sildenafil 25–50 mg or tadalafil 5–10 mg. HBOT for severe hypoxia or poor response. PGE1: anti-inflammatory, anti-platelet, pro-perfusive (evidence limited). CO₂ breathing techniques (optional; supportive vasodilation). Antibiotics: topical mupirocin; escalate if systemic signs. Analgesia: paracetamol ± ibuprofen; escalate to tramadol if required. Adrenaline only for true anaphylaxis. *[Free text]*

**Q8.** Reading the evidence, do you agree CMC gel, not PCL microspheres, is the primary contributor to acute obstruction in Ellansé® VO? *[Strongly Agree / Agree / Neutral / Disagree / Strongly Disagree]*

**Q9.** Based on the rheological data, a practical target is approximately 5 mL of saline per 1 mL of injected product (equivalent to a 1:5 dilution), delivered in repeated cycles until reperfusion is observed. *[Strongly Agree / Agree / Neutral / Disagree / Strongly Disagree]*

**Q10.** Timing should prioritise immediate flushing, followed by reassessment and possible repetition at 15–20 min intervals until reperfusion is restored. *[Strongly Agree / Agree / Neutral / Disagree / Strongly Disagree]*

**Q11.** Rigorous mechanical massage is required for effective CMC dispersion during flushing. *[Strongly Agree / Agree / Neutral / Disagree / Strongly Disagree]*

**Q12.** Ultrasound should be incorporated as a first-line tool (where available) for diagnosis and guided treatment in suspected VO. *[Strongly Agree / Agree / Neutral / Disagree / Strongly Disagree / Other]*

**Q13.** In some real-world settings, ophthalmology referral is a useful and viable option; however, <30 minutes is an unrealistic target. The terms used in the algorithm should be: *[Aim for critical 90-minute window / Aim for ‘as soon as possible’ / Keep 30-minute window as benchmark / Shorter: 15–20 minute window / Other]*

**Q14.** Retrobulbar/peribulbar injections for ocular emergencies done by experienced ophthalmologists should not be included in the algorithm. *[Strongly Agree / Agree / Neutral / Disagree / Strongly Disagree]*

**Q15.** Aspirin should remain the first-line adjunctive antiplatelet therapy; add LMWH (e.g., enoxaparin 40 mg SC daily or 1 mg/kg bid) as an escalation option in cases showing incomplete reperfusion or evolving necrosis despite standard measures. *[Strongly Agree / Agree / Neutral / Disagree / Strongly Disagree / Other]*

**Q16.** Any other comments? *[Free text]*
