# Supplementary material for: Staged Management of Vascular Occlusion With Ellansé (Polycaprolactone): A Modified Delphi Expert Consensus
Source: Aesthet Surg J Open Forum. 2026 Sep 30;8:ojag167. doi: 10.1093/asjof/ojag167 (PMC13625641; doi:10.1093/asjof/ojag167)
Supplement: Supplementary file 1 [file ojag167_supplementary_data.zip › Appendix 4.docx]

**APPENDIX 4: Consensus Process Details and Delphi Statements**

***Timeline***

The consensus process was initiated in May 2025 and completed in November 2025. Round 1 (anonymous survey) was distributed in May 2025, Round 2 (face-to-face meeting) was held in June 2025, and Round 3 (final anonymous survey) was completed in November 2025.

***Participation***

All seven invited panellists participated in all three rounds (response rate: 100%). Panellists were existing paid consultants and global trainers for Sinclair Pharma Ltd.; no additional financial incentives or honoraria were provided specifically for this exercise.

***Statement Evolution***

In Round 1, 30 statements were evaluated as part of an initial Scientific Advisory Board questionnaire assessing current clinical practice, safety considerations, and unmet needs. During Round 2, statements were refined, consolidated, and expanded based on panel feedback, with particular focus on VO recognition and management. In Round 3, 16 statements relating to VO management were formally assessed against predefined consensus criteria.

***Final Delphi Statements and Agreement Levels***
