# Supplementary material for: Staged Management of Vascular Occlusion With Ellansé (Polycaprolactone): A Modified Delphi Expert Consensus
Source: Aesthet Surg J Open Forum. 2026 Sep 30;8:ojag167. doi: 10.1093/asjof/ojag167 (PMC13625641; doi:10.1093/asjof/ojag167)
Supplement: Supplementary file 1 [file ojag167_supplementary_data.zip › Supplementary Figure 1.docx]

**Supplementary Figure 1. In vitro flow and dilution testing of PCL-CMC**

(A) Flow test, baseline (t=0): gel deposited at the top of each slide; experiment replicated 3 times. (B) Flow test (t=60 minutes): leading-edge migration is comparable for hyaluronidase (test) and water (control) slides; experiment replicated 5 times. (C) Dilution effect (1:1 and 1:2 water, t=15 minutes): marked increase in flow and loss of gel body; experiment replicated 3 times. Abbreviations: C, control solution; T, test solution.
