# Supplementary material for: Staged Management of Vascular Occlusion With Ellansé (Polycaprolactone): A Modified Delphi Expert Consensus
Source: Aesthet Surg J Open Forum. 2026 Sep 30;8:ojag167. doi: 10.1093/asjof/ojag167 (PMC13625641; doi:10.1093/asjof/ojag167)
Supplement: Supplementary file 1 [file ojag167_supplementary_data.zip › Supplementary Table 1.docx]

**Supplementary TABLE 1**: Literature Summary Table

*Summary of key studies that informed item generation for the modified Delphi consensus. Studies are grouped by topic area.*

| **Reference** | **Study Design** | **Topic / Key Findings** | **Relevance to Delphi Items** | **Item(s) Informed** |
| --- | --- | --- | --- | --- |
| Zaresharifi et al., 2025 | Case report | Management of PCL filler-induced VO; describes presentation and diagnostic challenges | Early recognition; management strategies for non-HA fillers | Recognition; First-line management |
| Guo et al., 2024 | Case report | Facial artery embolisation from PCL filler treated with multimodal regimen including 5-FU | Heterogeneity of management approaches; need for standardised protocol | Protocol structure; adjunctive therapies |
| Angelo-Khattar, 2022 | Clinical study | PCL microsphere persistence and slow hydrolytic degradation; minimal early mass loss | Supports CMC gel (not PCL microspheres) as primary acute VO driver | Mechanism statement |
| Murray et al., 2021 | Clinical guideline | Comprehensive HA filler VO management guideline (CMAC); staging, hyaluronidase protocol, adjunctive therapies | Framework for staging, recognition, adjunctive therapies; baseline for PCL-specific adaptation | Recognition; staging; aspirin; wound care |
| DeLorenzi, 2017 | Protocol/technique | High-dose pulsed hyaluronidase protocol for HA filler VO | Established enzymatic approach; inapplicable to PCL but informed dilution rationale | First-line dilution strategy |
| Madero-Pérez et al., 2025 | Comprehensive review | Guide for managing filler VO with cutaneous involvement; staging, vasodilator cautions, NTG exclusion | Adjunctive therapy hierarchy; vasodilator timing; nitroglycerin evidence review | Vasodilator strategy; NTG exclusion; second-line |
| van Loghem et al., 2020 | Expert consensus | Managing intravascular complications following CaHA treatment | Non-HA filler VO management principles; informed PCL extrapolation | Protocol framework |
| Kadouch et al., 2024 | Case series | US-guided hyaluronidase restored perfusion in CaHA/PLLA-induced VO despite no degradative effect | Rationale for retaining hyaluronidase in non-HA protocols | Hyaluronidase retention |
| Khan et al., 2024 | Preclinical (rabbit) | Heparin + nitroglycerin achieved ~80% perfusion recovery in PCL embolism; hyaluronidase ineffective | Supports non-enzymatic approach; informs anticoagulation and vasodilator discussion | Aspirin vs LMWH; NTG discussion |
| Buhsem et al., 2025 | Preclinical (rat) | PCL produced fewer lumen-filling thrombi than HA in rat model despite comparable endothelial injury | Lower thrombotic burden supports aspirin as proportionate first-line over LMWH | Aspirin vs LMWH |
| Alhusain et al., 2025 | SR/meta-analysis | 231 patients; early intervention (<6 h) improves outcomes; resolution rates for necrosis and visual impairment | Urgency of early intervention; time-critical management | Timing; reassessment intervals |
| Alam et al., 2021 | Systematic review | 77.1% VO risk reduction with cannulas vs needles | Prevention; injection technique recommendation | Pre-injection safety |
| Hwang et al., 2016 | Preclinical (rabbit) | No benefit and possible harm from topical NTG across four filler types; congestion observed | Counter-evidence for NTG; safety concern | NTG exclusion |
| Raia et al., 2022 | Clinical study | CRT kinetics after fluid challenge: detectable changes at 6–8 min, maximal at 10–12 min | Scientific basis for 15–20 min reassessment interval | Reassessment timing |
| *[Additional references to be completed]* |  |  |  |  |

*Note: This table summarises the principal studies that directly informed Delphi statement development. A complete bibliography is provided in the main manuscript reference list.*
