# Supplementary material for: Staged Management of Vascular Occlusion With Ellansé (Polycaprolactone): A Modified Delphi Expert Consensus
Source: Aesthet Surg J Open Forum. 2026 Sep 30;8:ojag167. doi: 10.1093/asjof/ojag167 (PMC13625641; doi:10.1093/asjof/ojag167)
Supplement: Supplementary file 1 [file ojag167_supplementary_data.zip › Supplementary Table 2.docx]

**Supplementary Table 2**

| **Component** | **Statement** | **Consensus** |
| --- | --- | --- |
| Mechanism | CMC carrier gel is primary contributor to acute obstruction (vs PCL microspheres) | Full |
| Preparation | Emergency kit, baseline CRT, escalation planning | Full |
| Recognition | CRT is primary diagnostic/monitoring parameter | Full |
| Recognition | Reassess CRT during management at 15–20 min intervals | Strong (≥80%) |
| Recognition | Pain and skin colour/changes as additional diagnostic indicators | Full |
| Recognition | Compare perfusion with adjacent normal tissue | Strong (≥80%) |
| Recognition | Ultrasound (where available) for localisation and guided treatment | Partial (≥70%) |
| Ocular | Visual symptoms: urgent ophthalmology/stroke service referral ASAP | Full |
| First-line | Immediately stop injection on suspected VO | Full |
| First-line | Saline flushing + hyaluronidase (adjunctive) + immediate massage | Strong (≥80%) |
| First-line | Hyaluronidase retained despite no degradative effect | Strong (≥80%) |
| First-line | Target dilution ≈5 mL saline per 1 mL product (≈1:5) | Full |
| First-line | Vigorous massage ≥2 minutes per locale after flushing | Strong (≥80%) |
| First-line | Reassess every 15–20 min; repeat cycles until reperfusion | Strong (≥80%) |
| First-line | Aspirin 300 mg loading then 75 mg daily as first-line above LMWH | Partial (≥70%) |
| Second-line | Pentoxifylline 400–800 mg BD for 7 days | Strong (≥80%) |
| Second-line | Corticosteroids for marked inflammation (avoid routine use) | Strong (≥80%) |
| Adjunctive | Avoid vasodilators in acute phase | Partial (≥70%) |
| Adjunctive | If used post-reperfusion: prefer CCBs over PDE-5 inhibitors | Undetermined |
| Adjunctive | HBOT (if available) for severe hypoxia/poor response | Partial (≥70%) |
| Ocular | Exclude retrobulbar/peribulbar injections from algorithm | Partial (≥70%) |
